# Association of endogenous progesterone levels in young women using hormonal contraception with recent HIV-1 infection

**DOI:** 10.1186/s12905-019-0761-y

**Published:** 2019-05-08

**Authors:** Resha Boodhram, Dhayendre Moodley, Nathlee Abbai, Gita Ramjee

**Affiliations:** 1 HIV Prevention Research Unit, South African Medical Research Council, Durban, South Africa; 2 Department of Obstetrics and Gynaecology, School of Clinical Medicine, College of Health Sciences, University of KwaZulu Natal, 719 Umbilo Road, Durban, 4051 South Africa; 3 Department of Clinical Medicine Laboratory, School of Clinical Medicine, College of Health Sciences, University of KwaZulu Natal, Durban, South Africa; 4 Department of Epidemiology and Population Health, London School of Hygiene & Tropical Medicine, London, UK; 5 Department of Global Health, University of Washington, Seattle, USA

**Keywords:** Hormonal contraception, Endogenous progesterone, Human immunodeficiency virus acquisition

## Abstract

**Background:**

A high endogenous progesterone luteal state in the menstrual cycle has been independently associated with Human Immunodeficiency Virus (HIV) incidence in epidemiological studies. Hormonal contraception particularly high dose Depot Medroxyprogesterone Acetate (DMPA) is also thought to increase the risk of HIV acquisition. Inconsistent reports of this association have led us to hypothesize that unsuppressed endogenous progesterone level in women who reported hormonal contraception (HC) use may be an explanation for increased vulnerability to HIV.

**Methods:**

This pilot study was a secondary cross-sectional analysis of data and laboratory testing of stored specimens collected from women who participated in the SAMRC HIV prevention MDP 301 trial during 2005–2009 in South Africa. Serum progesterone levels were measured in 39 women at the point of first positive HIV diagnosis during study follow-up and 36 women who remained HIV uninfected at the 52-week study exit visit.

**Results:**

Overall, the median (IQR) progesterone level in 49 women using hormonal contraception was 0.39 ng/ml (IQR 0.13–0.45) and 48 (97.9%) women had a progesterone level < 3.0 ng/ml suggestive of adequate progesterone suppression for contraceptive efficacy. After excluding the one woman with a progesterone level of > 3.0 ng/ml, the median progesterone level in women using DMPA remained marginally higher at 0.42 ng/ml (IQR 0.27–0.45) than women using Norethisterone Enanthate (NET-EN) (0.31 ng/ml; IQR 0.13–0.41, *p* = 0.061). For women using hormonal contraception, the median progesterone level did not differ between women with recent HIV infection or women who remained HIV negative (0.39 vs 0.38 ng/ml, *p* = 0.959). Similarly, the median progesterone level in women using DMPA or NET-EN did not differ by HIV status (0.43 vs 0.41 ng/ml, *p* = 0.905; 0.24 vs 0.31 ng/ml, *p* = 0.889).

**Conclusion:**

Among women using hormonal contraception, DMPA or NET-EN we did not observe a significant difference in progesterone levels between women with recently acquired HIV infection and women who remained HIV negative. Our findings suggest that endogenous progesterone levels remain suppressed in the presence of hormonal contraception and are not likely to be associated with HIV acquisition.

## Implications

In one of a handful of studies in women, our findings are suggestive that the endogenous progesterone level remains adequately suppressed in the presence of high dose HC and may not be associated with recently acquired HIV acquisition. If a high endogenous progesterone state in women not on HC has been implicated in HIV acquisition as suggested in our study as well as several others, it is quite likely that the high Medroxyprogesterone Acetate (MPA) (exogenous progesterone) level following a high dose DMPA administration could hypothetically and independently increase susceptibility to HIV acquisition. There is a need for more studies to measure MPA levels before and after HIV seroconversion and soon after a high dose DMPA administration.

## Background

An estimated 7 million South Africans are living with Human Immunodeficiency Virus (HIV) and women aged 15 and older constitute more than 57% of those affected [1]. In comparison to their male counterparts, young women are disproportionately affected by incident HIV infections [2]. Acquisition of HIV infection in young women has been associated with a range of behavioural risk factors, demographic characteristics and biological factors [3–5]. Biological risk in the general population includes altered host immune responses and the presence of other sexually transmitted infections (STI) [6–8]. Hormonal control of mucosal immune responses and integrity of the genital mucosal barrier have also been implicated in increased susceptibility to HIV acquisition in women [9]. In-vitro experimental studies have demonstrated that the hormone progesterone promotes cell susceptibility to HIV-1 and enhances HIV replication in infected cells by altering inflammatory cytokine responses [10, 11]. More specifically, Cabrera-Munoz et al.,(2012) demonstrated that progesterone plays a role in modulating CCR5 and CXCR4 expression in HIV infected and uninfected women [10]. Evidence of increased vulnerability to HIV during the luteal phase of the menstrual cycle, during pregnancy, lactation and menopause are also suggestive of an association between high endogenous progesterone levels and altered mucosal immunity during these progesterone dominant periods [12–14].

Findings from epidemiological studies have also implicated progestin-only Depot Medroxyprogesterone Acetate (DMPA), a form of exogenous progesterone, as a potential risk for HIV acquisition and have attributed a 40% increase in HIV incidence to users of DMPA in comparison to women not using hormonal contraception or users of Norethisterone Enanthate (NET-EN) [15, 16]. A study conducted by Byrne et al. (2016) in Umlazi, South Africa found that the use of injectable progestin-only contraceptive and high endogenous progesterone levels were both independently associated with increased frequency of activated HIV targets cells at the cervix [17].

We hypothesized that unsuppressed endogenous progesterone levels in women using hormonal contraception are likely associated with recently acquired HIV infection. In addition, we also determined if there was an association between serum progesterone level in women not on hormonal contraception and recently acquired HIV infection.

## Methods

This study was a retrospective analysis utilizing stored serum samples from women who had participated in the Microbicides Development Programme (MDP) 301 trial conducted between 2005 and 2009 [18]. The trial was an international, multicenter, randomized, double-blind, placebo-controlled trial to assess the safety and efficacy of 0.5 and 2% PRO 2000/5 microbicide gels for the prevention of vaginally acquired HIV infection. A full description of the parent study has previously been published [18]. Women were tested for HIV-1 at 3-month intervals. HIV status was determined using the Abbott Determine HIV1/2 Rapid HIV test kit and Recombigen Unigold HIV-1 Rapid test kit. Discordant and positive HIV rapid test results were confirmed with HIV Enzyme-Linked Immunosorbent Assay (ELISA). The methods of contraception used by women in the parent study were DMPA, NET-EN, oral contraceptives and non-hormonal methods (tubal ligation, male condoms, female condoms, natural methods, and traditional methods). The HIV incidence in the MDP 301 trial was reported as 4.5 (95%CI 3.8–5.4) per 100 woman years for the active gel versus 4.3 (95%CI 3.6–5.2) per 100 woman years for placebo. In the latter study, 0.5 and 0.2% Pro2000 vaginal gel was not efficacious in preventing HIV-1 acquisition.

For this study, we adopted a convenience sampling approach based on the availability of adequate stored specimen volume needed for determining the serum progesterone level. Adequate specimens were available for 39 women who seroconverted (HIV-1 seropositive) during the course of the parent study and 36 women who remained HIV uninfected at the end of the parent study (week 52). Samples were obtained from the first diagnosis of seroconversion and at the last study visit for women who remained HIV uninfected. Informed Consent (IC) was provided for long term storage and future testing.

The study was reviewed by the Biomedical Research Ethics Committee (BREC) of the University of KwaZulu-Natal and full ethical approval granted on 16 September 2015 (BE352/15).

Serum progesterone testing was performed at the HIV Prevention Research Unit of the SAMRC in Durban, South Africa. Serum samples that were stored at -70 °C for future testing were utilized to measure the progesterone levels. A total of 200 μL of serum was used for testing. The VIDAS® Progesterone (PRG) assay which is an enzyme-linked fluorescent immunoassay (ELIFA) was used with the VIDAS (VITEK® ImmunoDiagnostic Assay System) instrument as per manufacturer’s instructions. The measurement range of the VIDAS Progesterone reagent was 0.25 - 80 ng/ml. Progesterone values below the limit of detection (< 0.25 ng/ml) were read as half the limit of detection (i.e. 0.125 ng/ml). The range for the follicular phase was < 0.25–0.54 ng/ml and luteal phase was 1.5–20 ng/ml as per the Mini VIDAS package insert. We added the intermediate phase if serum progesterone levels were between 0.54 and 1.5 ng/ml. Only women who were not on contraception were grouped into “Phase of Menstrual Cycle” according to the defined progesterone level in the Mini VIDAS package insert.

## Results

In this select study population, for whom we had an adequate stored specimen for measuring progesterone level, the demographic and behavioural characteristics were comparable between women with recently acquired HIV infection and women who remained HIV negative at end of study follow-up (Table 1). The majority (65.3%) of women were using hormonal contraception and DMPA (63.3%), as opposed to NET-EN was the more common choice for contraception. The median length of follow-up of women selected for this sub-study was 52 weeks. The median time to the first HIV positive test since enrolment was 24 weeks (IQR 12–52).

**Table 1:** Demographic and behavioural characteristics of women with recent HIV infection and women who remained HIV negative at the end of study follow-up

	HIV Positive *n* = 39	HIV Negative *n* = 36	Total *n* = 75	*P* value
Age				0.819
< 25	21 (53.8%)	18 (50.0%)	39 (52.0%)	
> 25	18 (46.2%)	18 (50.0%)	36 (48.0%)	
Education				1.000
None	28 (71.8%)	25 (69.4%)	53 (70.7%)	
Some form of Education	11 (28.2%)	11 (30.6%)	22 (29.3%)	
Sexual Debut				0.129
< 19 years	35 (89.7%)	27 (75.0%)	62 (82.7%)	
> 20 years	4 (10.3%)	9 (25.0%)	13 (17.3%)	
*Number of Sex Acts in the last 7 days				1.000
1–2	16 (55.2%)	16 (57.1%)	32 (56.1%)	
3+	13 (44.8%)	12 (42.9%)	25 (43.9%)	
◊Any STI				0.389
No	33 (84.6%)	27 (75.0%)	60 (80.0%)	
Yes	6 (15.4%)	9 (25.0%)	15 (20.0%)	
Hormonal Contraception				0.628
No	15 (38.5%)	11 (30.6%)	26 (34.7%)	
Yes	24 (61.5%)	25 (69.4%)	49 (65.3%)	
Type of Hormonal Contraception				0.560
DMPA	14 (58.3%)	17 (68.0%)	31 (63.3%)	
NET-EN	10 (41.7%)	8 (32.0%)	18 (36.7%)	

◊Syphilis, Trichomonas vaginalis, Neisseria gonorrhoeae, Chlamydia trachomatis

Serum samples for all 75 participants yielded valid results for progesterone levels. The median progesterone level in 39 women with recently acquired HIV infection did not differ significantly from the 36 women who remained HIV negative at study end (0.40 ng/ml versus 0.34 ng/ml, *p* = 0.296). Overall, the median (IQR) progesterone level in 49 women using hormonal contraception was 0.39 ng/ml (IQR 0.13–0.45) and 48 (97.9%) women had a progesterone level < 3.0 ng/ml suggestive of adequate progesterone suppression for contraceptive efficacy. After excluding the one woman with a progesterone level of > 3.0 ng/ml, the median progesterone level in women using DMPA remained marginally higher at 0.42 ng/ml (IQR 0.27–0.45) than women using NET-EN (0.31 ng/ml; IQR 0.13–0.41, *p* = 0.061) (Table 2).

**Table 2:** Endogenous progesterone level (ng/ml) in women using hormonal contraception in association with recently acquired HIV infection

	HIV Positive	HIV Negative	*P* Value
Hormonal Contraception (*n* = 49)	*n* = 24	*n* = 25	0.959
Median (IQR) ng/ml	0.39 (0.13–0.44)	0.38 (0.13–0.45)	
No Contraception (*n* = 26)	*n* = 15	*n* = 11	0.122
Median (IQR) ng/ml	0.44 (0.31–4.79)	0.27 (0.13–0.74)	
DMPA (*n* = 31)	*n* = 14	*n* = 17	0.905
Median (IQR) ng/ml	0.43 (0.32–0.45)	0.41 (0.27–0.46)	
NET-EN (*n* = 18)	*n* = 10	*n* = 8	0.889
Median (IQR) ng/ml	0.24 (0.13–0.44)	0.31 (0.13–0.38)	

In the presence of hormonal contraception, none of the 24 women with recent HIV infection and one of the 25 HIV negative women had an unsuppressed level of progesterone (> 3.0 ng/ml), the maximum concentration for contraceptive effectiveness [19].

Stratified by HIV status, median progesterone levels did not differ among women using hormonal contraception (0.39 vs 0.38 ng/ml), or not using contraception (0.44 vs 0.27 ng/ml), or using either DMPA (0.43 vs 0.41 ng/ml) or NET-EN (0.24 vs 0.31 ng/ml) (Table 2).

Among the 15 women with recent HIV infection and were not using hormonal contraception, 4 (26.7%) were categorized as being in the luteal phase, compared to one among the 11 HIV negative women (9.1%) (*p* = 0.356). The serum progesterone ranges for the luteal, intermediate and follicular phases were 7.73 ng/ml, 0.58–1.24 ng/ml and 0.13–0.27 ng/ml respectively.

## Discussion

In the presence of hormonal contraception (DMPA or NET-EN), the lack of an association between endogenous progesterone levels and recently acquired HIV infection in our sub-study is suggestive of other potential mechanisms of risk for HIV acquisition associated with DMPA. Although we observed more naturally cycling women with recent HIV infection to be in the luteal phase, a period of high endogenous progesterone level, the small sample size and the timing of blood draws in respect to the actual time of HIV acquisition were limiting in demonstrating an association between high endogenous progesterone level and HIV acquisition.

Contrary to our hypothesis that higher levels of endogenous progesterone in the presence of hormonal contraception could be implicated in HIV acquisition, it appears that synthetic progestins such as MPA in this population were maintained at an adequately high level associated with the suppression of endogenous production of progesterone [20]. In other words, our findings are also suggestive that women included in this sub study and who self-reported hormonal contraception use, were indeed using hormonal contraception. Unfortunately, we did not measure exogenous progestin (MPA) level in these women, neither did we have data describing the timing of the last administration dose of HC prior to sampling. Animal studies have revealed a thinning of the vaginal epithelium and an increase in CD4+ target cell population in the squamous epithelium associated with a high dose of DMPA treatment [21–23]. And the presence of virions in genital tissue during this high progestin state is supporting evidence that high levels of exogenous progestin could likely be implicated in HIV acquisition in women [23]. It was not the intention of this sub study to determine the association of hormonal contraception and HIV acquisition, however a secondary analysis of data from the parent study that included 382 seroconversions and more than 8000 HIV negative women, demonstrated a higher risk of HIV acquisition (HR 1.49) among women who used DMPA as compared to women on NET-EN and oral contraception [24]. The authors do however caution on a limitation of these findings as this was a secondary analysis and residual confounders could not be excluded. So the question remains, could the women on DMPA and acquired HIV have higher exogenous progestin levels than women also on DMPA but remained HIV uninfected?

When stratifying the data according to phases of the menstrual cycle in non-HC users, for women who were considered to be in the luteal phase, the results showed a two-fold higher level of serum progesterone in women who seroconverted versus women who remained HIV negative. Yet again, there is conflicting evidence of the association between the high-progesterone state during the luteal phase of the menstrual cycle and HIV acquisition. Byrne et al. (2016) measured plasma progesterone levels and found that women who did not use HC had a 3.25 times higher frequency of cervical target cells in the luteal phase of the menstrual cycle than those in the follicular phase (*p* = 0.04) thus suggesting endogenous progesterone levels are associated with HIV acquisition [17]. Another study showed that leukocyte infiltration and proteins involved in tissue remodelling with the luteal phase could potentially increase women’s susceptibility to HIV infection [12]. In macaque models, Carias et al. (2016) provided more evidence of dynamic changes in vaginal epithelial structure and CD4+ target cell presence associated with both high endogenous and exogenous progesterone/progestin-dominant phases [21].

### Limitations

We acknowledge that the small sample size of the study was a limiting factor in drawing sound conclusions. This was a retrospective analysis of data and therefore subjected to limited data availability such as the timing of administration of injectable HC. In addition, HIV testing was performed at 3-month intervals and we depended on the first diagnosis of seroconversion and not the actual time of HIV acquisition noting that women not on HC would have had a 28-day menstrual cycle and women on HC would have had a 3-monthly (DMPA) or 2-monthly (NET-EN) administration of HC. Essentially, blood samples were collected sometime after HIV acquisition. And more importantly, we did not measure synthetic progestin-level (MPA) in women on HC (DMPA and NET-EN).

## Conclusion

Our findings suggest that the endogenous progesterone level in the presence of hormonal contraception is adequately suppressed and may not be the reason for the higher risk of HIV acquisition associated with hormonal contraception, particularly DMPA.

## Abbreviations

DMPA: Depot medroxyprogesterone acetate
HC: Hormonal contraception
HIV: Human immunodeficiency virus
IC: Informed consent
MPA: Medroxyprogesterone acetate
NET-EN: Norethisterone enanthate
SAMRC: South African medical research council
STI: Sexually transmitted infection
